# Survival of severely compromised endodontically treated teeth restored with or without a fiber glass post

**DOI:** 10.1590/1678-7757-2023-0241

**Published:** 2023-10-27

**Authors:** Maria Tereza Hordones RIBEIRO, Gabriella de OLIVEIRA, Helena Letícia Quirino de OLIVEIRA, Lilibeth Carola Leyton MENDOZA, Calebe de MELO, Thiago Silva PERES, Carlos José SOARES

**Affiliations:** 1 Universidade Federal de Uberlândia Faculdade de Odontologia Departamento de Dentística e Materiais Odontológicos Uberlândia Minas Gerais Brasil Universidade Federal de Uberlândia, Faculdade de Odontologia, Departamento de Dentística e Materiais Odontológicos, Uberlândia, Minas Gerais, Brasil.

**Keywords:** Post and core technique, Fatigue, Endodontically-treated teeth, Composite resins

## Abstract

**Objective:**

The use of a fiber glass post (FGP) type and choice of FGP diameter to restore endodontically treated incisors without ferrule is controversial. This study evaluated survival rate and failure mode of severely compromised central incisors without ferrule rehabilitated using resin-based composite (RBC) with or without FGP with different diameters.

**Methodology:**

A total of 60 decoronated bovine incisors without a ferrule were endodontically treated and prepared for 1.4, 1.6, and 1.8 mm diameter FGPs (Whitepost System DC 0.5, Fit 0.4, and DCE 0.5; FGM). Half of the teeth received FGPs cemented using dual-cure resin cement (Allcem Core; FGM), the other half were filled using only bulk-fill RBC (OPUS Bulk Fill; FGM). The crowns were directly restored with RBC. The roots were embedded in polystyrene resin and the periodontal ligament was simulated with polyether impression material. Fatigue testing was conducted under 5 Hz cyclic loading at 30 degrees to the incisal edge, beginning at 50 N (5,000 cycles) as a warmup. After, the load was increased 100 N every 15,000 cycles until fracture occurred. All specimens were subjected to transillumination, micro-CT analysis, and digital radiography before and after fatigue testing. Fracture mode was classified according to severity and repair potential. Data were analyzed with Kaplan-Meier survival test and post hoc log-rank test (α=0.05) for pairwise comparisons.

**Results:**

Using FGP significantly increased the number of cycles to failure, irrespective of FGP diameters (p=0.001). The FGP diameters had no statistically significant effect on cycles to failure or failure mode.

**Conclusion:**

Using FGP without ferrule improved survival rate of structurally severely compromised central incisors compared with rehabilitation without FGP. The diameter of the FGPs had no effect on the survival rate and failure mode.

## Introduction

Root canal treatment is a common clinical routine in dentistry. At least one adult out of two has one tooth with canal treatment.^1^ Many endodontically treated teeth lack sufficient structure to retain a restoration or crown.^2,3^ Ferrule could improve retention for the restorative material.^3-5^ However, in many situations, the amount of coronal structure is outside the control of the clinician due to caries, dental trauma, or previous restorations.^5,6^

Fiber glass posts (FGPs) provide the needed retention in cases without coronal structure. However, the literature on its benefits is divided.^7,8^ Recent laboratory studies suggest that using FGP is unnecessary for restoring teeth, regardless of a ferrule presence, and may generate more catastrophic fractures.^9-11^ Nonetheless, clinical studies reported improved survival rates with a FGP in the presence and absence of ferrule.^12-14^ Some laboratory and computational studies also demonstrated that a FGP could improve the strength of restored teeth,^15-17^ when adhesive cemented into the canal, creating better stress distribution.^18-20^ The stress and strain, related to indication or not of FGPs, are more challenging for restoring anterior teeth due to the lateral component forces during the masticatory process.^2,4^

Another controversial issue is the best diameter for a FGP.^21-24^ Mechanical properties of a FGP are determined by the type, size, density, and distribution of fibers and their bond with the matrix.^25,26^ Generally, thicker FGPs can be expected to be stronger,^24^ but the effect of increasing the thickness may not offer better benefits concerning retention and long-term survival of the restoration.^21,22^ Additionally, to increase a FGP diameter, more root canal dentin must be sacrificed.^27,28^ A conservative dentistry concept would rather adapt the size of a FGP to the size of the canal. The restorative procedure should follow the same principle, selecting the FGP that best fits the prepared root canal. The new FGPs concept, like the Whitepost System FIT 0.4 (FGM), has been designed to follow the same conservative principles performed during endodontic treatment. To the author’s knowledge, no study has tested the mechanical performance of this type of FGP compared with conventional smooth dual conic FGP.

Therefore, this study aimed to evaluate the survival rate and failure mode of relatively thin FGP systems designed according to a conservative principle and compare them to a bulk-fill resin-based composite (RBC) root canal foundation without FGP. Three FGP diameter sizes will be tested for restoring severely compromised central incisors without ferrule. The two null hypotheses were that 1) the presence of FGP and the use of the bulk-fill RBC foundation without FGP and 2) the diameter of the FGP do not influence the survival rate and the failure mode of severely compromised central incisors without ferrule restored with an RBC crown.

## Methodology

### Specimen preparation

A total of 60 bovine incisors with straight roots and similar dimensions were selected and stored in distilled water at 4°C until use. The crowns were removed with a double-sided diamond disc (Discoflex, KG Sorensen, Barueri, SP, Brazil) at low speed with air/water cooling spray, leaving 15 mm of the root. To simulate the periodontal ligament, a 0.3 mm layer of polyether impression material (Impregum F, 3M Oral Care, St Paul, MN, USA) was applied to cover the roots, and embedded in a polystyrene resin (Cristal, Piracicaba, SP, Brazil) until reaching a depth of 2 mm below the cemento-enamel junction, replicating the alveolar bone.^29^

The root canals were treated using 25 mm rotary files (Trunatomy #36.03, Dentsply Sirona, Charlotte, NC, EUA). Motor rotation was regulated at 500 rpm and 1.5 Ncm torque. Apical patency was maintained with a #30 hand file. Root canal irrigation was performed using 2.5% sodium hypochlorite (Asfer, São Caetano do Sul, SP, Brazil) with a syringe and endodontic needle (Navitip, Ultradent, South Jordan, UT, USA), while the solution was absorbed with endodontic suctor (Flex Suctor, Angelus, Londrina, PR, Brazil).^30^ The root canal was cleaned with 17% ethylenediaminetetraacetic acid (EDTA, Maquira, Maringá, PR, Brazil), activated using cleaning files (Easy Clean, Dentsply Sirona). Final irrigation was performed with distilled water, followed by drying the canals with paper cones #40 (Absorbent Paper Points, Dentsply Sirona). Obturation was carried out with gutta-percha accessory cones (M size Guttapercha Accesory Cone, Dentsply Sirona) and endodontic resin cement (AH Plus, Dentsply Sirona).

All groups had gutta-percha removed 10 mm deep and 5 mm was left apically. Then, the canals were instrumented using drills No. 1 and 2 (Gates Drills, DiaDent, Burnaby, BC, Canada) in combination with 17% ethylenediaminetetraacetic acid (EDTA, Maquira) irrigation activated with a flexible instrument (EasyClean, Easy Equipment, Belo Horizonte, MG, Brazil).

**Figure 1:**
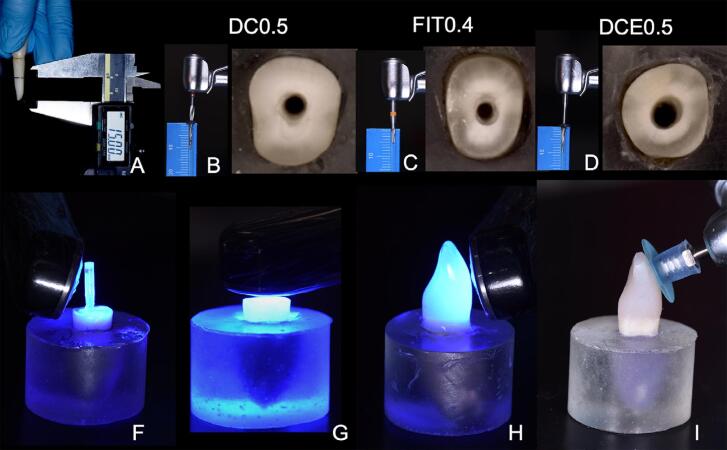
Sequence of specimen preparation for groups with or without fiber glass post (FGP). A, bovine incisor delimitation of root length; B, DC0.5 root canal preparation using specific drill with 10.0 mm length; C, FIT0.4 root canal preparation using specific drill with 5.0 mm length; D, DCE0.5 root canal preparation using thicker specific drill with 10.0 mm length; E, light curing of dual-cure resin cement used to cement in FGP groups; F, light curing bulk-fill resin-based composite (RBC) inside root canal for groups without a FGP; G, light curing bulk-fill RBC with transparent crown matrix; H. Finishing and polishing of completed specimen

**Figure 2:**
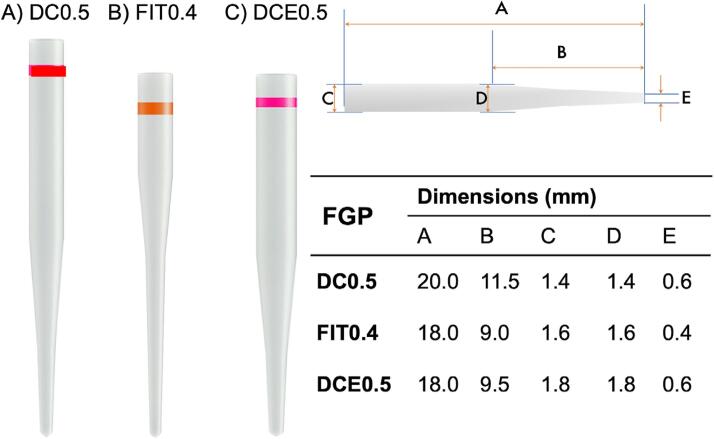
Dimensions of the Whitepost System fiber glass posts used; A. DC0.5; B. FIT0.4; and C. DCE0.5

The mounted roots were assigned to three groups, each prepared with a different drill representing a different diameter of a dual conic/cylindrical FGP system (Whitepost System, FGM, Joinvile, SC, Brazil): 1) 1.4 mm (DC 0.5); 2) 1.6 mm (Fit 0.4); and 3) 1.8 mm (DCE 0.5). (Figures 1 and 2). The 1.6 mm drill prepared only the cervical third of the root, whereas the 1.4 and 1.8 mm drills prepared the root canal up to 10 mm deep. The canal was irrigated with distilled water during this procedure to avoid heat generation, and dried with paper cones (Absorbent Paper Points, Dentsply Sirona) afterwards.

After root canal preparation, the adhesive system (Ambar Universal APS, FGM) was applied into the root canal with a brush (Cavibrush, FGM) and on the flat coronal root surface, followed by gentle air spray. Excess was removed with a paper cone. The adhesive was photo-activated for 20 s using a light-curing unit (VALO Grand, Ultradent) with 939 mW/cm^2^, measured with an integrating sphere (Labsphere, North Sutton, NH, USA) connected to a fiber-optic spectroradiometer (USB 4000, Ocean Insight, Rochester, NY, USA).

**Figure 3:**
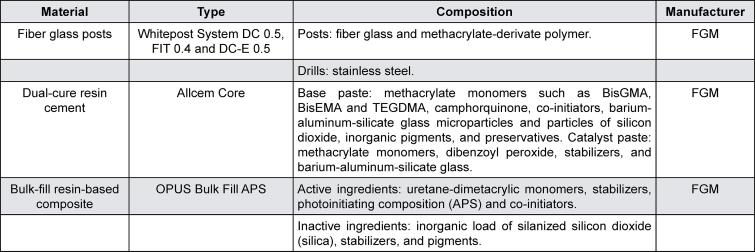
Materials and manufacturer information

The FGP was cleaned with 70% alcohol (Asseptagel, Start, Uberlândia, MG, Brazil). Silane (Prosil, FGM) was applied for one minute followed by air spray application. The canal was filled with automix resin cement (Allcem Core, FGM) using an endo tip.^31^ The specific FGPs for each group were inserted into the root canals with digital pressure, and resin cement excess was removed. After waiting for 5 min the resin cement was photo-activated for 40 s on incisal, buccal, and lingual faces.^32^Figure 3 shows the information from the materials provided by the manufacturers.

For the groups without FGP, bulk-fill RBC (OPUS Bulk Fill APS, FGM) was inserted into the root canal in two 5 mm increments. The bulk-fill RBC was condensed to prevent air bubbles. Each increment was photo-cured for 40 s. Standardized transparent central incisor plastic acetate matrix (Coroas Refil, TDV, Pomorode, SC, Brazil) with 11 mm cervical-incisal dimension, was filled using the same bulk-fill RBC in a single increment and was adapted to the root using digital pressure. After removing excess, the bulk-fill RBC was photo-cured for 40 s from buccal, incisal, and lingual directions with the VALO Grand light curing unit. The matrix was removed with a scalpel blade (Advantive, Wuxi Xinda Medical Device, Jiangsu, China) and the bulk-fill RBC was finished and polished with aluminum-oxide discs (Diamond discs, FGM).

### Fatigue load test

The specimens were submitted to a 5 Hz cyclic fatigue load using an electrodynamic testing machine (Eletropulse E3000, Instron, Norwood, MA, USA). The force was applied simulating masticatory forces to incisal edge at an angle of 30 degrees with the flat surface covered with RBC.^33^ A cyclic load of 50N was applied at a 5Hz, beginning with a warmup period of 5,000 cycles. After the first 5,000 cycles, if the specimen had not failed, the maximum load was increased by 100 N every 15,000 cycles until failure occurred.^9^ The specimens remained submerged in distilled water at 37°C during the fatigue test. All tests were filmed to identify the initial and final failure cycles.

### Transillumination

**Figure 4:**
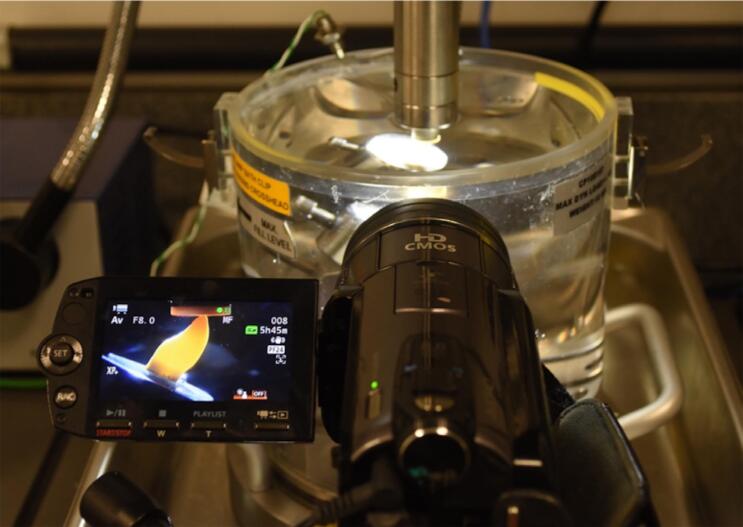
Macro camera recording the specimen in load chamber using transillumination to identify initial and final failure

**Figure 5:**
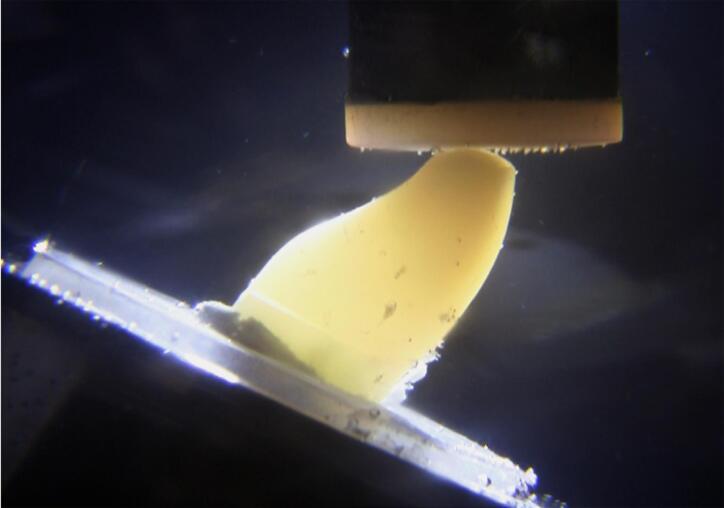
Fatigue loading applied to incisal edge at 30° angle. Initial failure can be seen by lingual gap between bulk-fill resin-based composite and tooth structure

**Figure 6:**
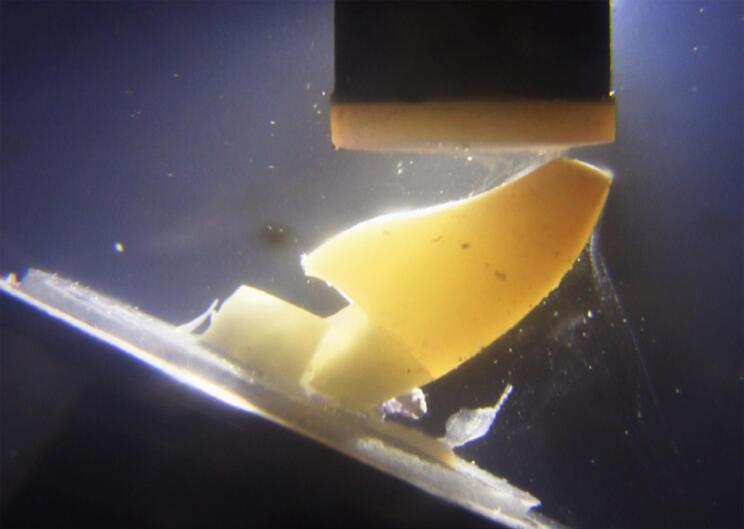
Final failure after fatigue loading, demonstrating buccal root fracture invading the limit of simulated bone support

Transillumination was conducted during the fatigue load test using a macro video camera (Vixia HF S100, Canon, Nagasaki, Japan) and a LED light (P1050, Photonita, Florianópolis, SC, Brazil) in the background to detect where the initial failure occurred (Figure 4) and the test was continuously recorded until final failure (Figures 5 and 6). Specimens were evaluated to detect and classify the fracture mode using transillumination, micro-CT analysis, and digital radiography. The images of specimens were captured under standardized conditions, and were taken with the EOS camera (Nikon D7200, Nikon, Tokyo, Japan) at the same settings (ISO 200, f/18, 1/200 s). The transillumination light (P1050, Photonita) was used with the optic fiber illuminator positioned on the tooth’s lingual surface.

### Micro-CT analysis

Specimens were scanned in a microcomputed tomography (micro-CT) device (SkyScan 1272, Bruker, Kontich, Belgium) at two instances: before starting the accelerated fatigue and after the final failure.^31,34^ Scanning was conducted under the following conditions: 100 KV and 100 μA, 20 μm pixel size, 2000 ms exposure time, 180° rotation angle at a rotation step of 0.4, frame averaging of 2, random movement of 30, and a Cu filter of 0.11 mm. The images acquired by micro-CT were reconstructed by a NRecon software program (version 1.6.3.3; Bruker micro-CT) with a beam hardening correction of 2%, a smoothing level of 1, and a ring artifact correction level of 7. A Data Viewer (version 1.14.4.1 SkyScan, Bruker) was used to identify the region corresponding to the initial failure and final break, obtaining axial, sagittal, and transverse slices of 3D volume before and after the failure fatigue process.

### Digital radiographs

**Figure 7:**
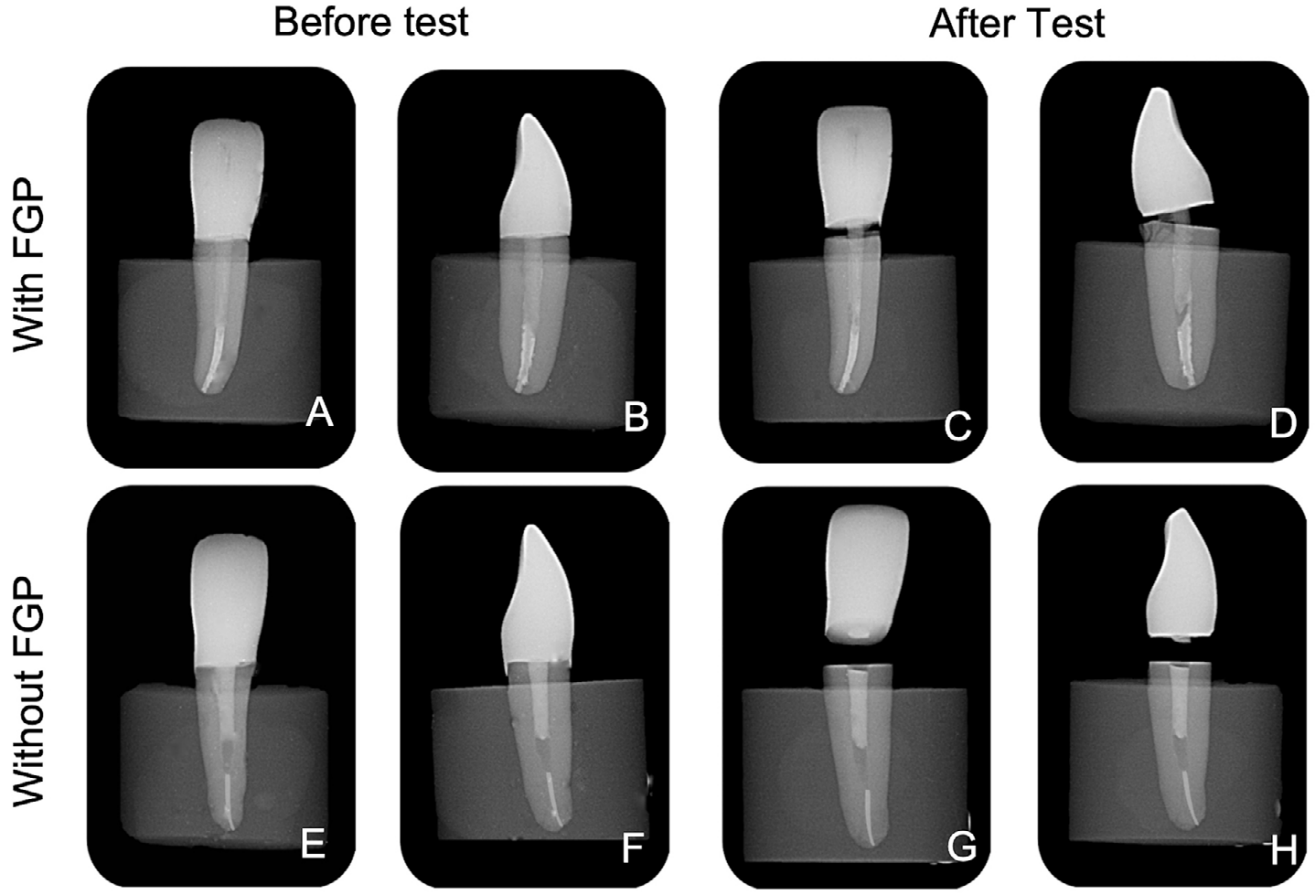
Digital frontal and lateral radiographs of the specimens before and after fatigue testing. A, Specimen restored with fiber glass post (FGP) demonstrating no bubbles and adaptation of the resin cement layer; B, Lateral image demonstrating the residue of root filling material at apical third, and also good adaptation of FGP along the root canal; C, Same specimen after fatigue test with partial crown displacement without FGP fracture; D, Lateral image after fatigue test demonstrating FGP debonding and root dentin fracture at the cervical limit; E, Specimen restored without FGP demonstrating a large bubble at apical limit resin-based composite retention; F, Lateral image demonstrating the residue of root filling material at apical third, and good adaptation of FGP along the root canal; G, Same specimen after fatigue test with partial crown displacement without FGP fracture; H, Lateral image after fatigue test demonstrating FGP debonding and root dentin fracture at the cervical limit

Digital radiographs were also taken before and after the fatigue tests (Figure 7). The specimens were positioned on a matched plate and radiographic exposure was performed with radiography equipment (Timex 70 E, Gnatus, Ribeirão Preto, SP, Brazil) exposing the tooth 0.25 s at 70 kV and 7.0 mA. The focal length was 50 cm. The radiographs were transferred from the phosphor plate (size 2, Durr Dental) to the computer by using a scanner (Vistascan, Durr Dental, Bietigheim-Bissingen, Germany).

### Failure mode analysis

The images from transillumination, micro-CT analysis, and digital radiographs were uploaded onto a computer, and three calibrated operators determined the fracture mode in a blinded process.

The fracture mode at final failure was classified using five categories of reparable (I-III), possibly reparable (IV), and catastrophic fractures (V):

I – Failure of the adhesive interface without displacement of the crown and without involvement of tooth structure – Repairable;
II – Crown fracture without post fracture/retention and without tooth structure involvement – Repairable;
III – Cervical crown fracture with post/retention breakage without tooth structure involvement – Repairable;
IV – Crown/post fracture involving repairable tooth structure – Possibly repairable;
V – Crown/post fracture with involvement of tooth structure requiring extraction – Catastrophic.

### Statistical analysis

The fatigue resistance (based on number of cycles to failure) among the groups was compared with the Kaplan-Meier survival estimator for load cycles to initial and final failures. A *post hoc* log-rank test was used for pairwise comparisons among the six groups and between the initial and final failure within each group (corrected for multiple comparisons when indicated). The fracture mode frequency was analyzed using Chi-square test. All tests used a significance level of α=0.05. The statistical analysis was performed using SPSS software (IBM SPSS Statistics v23, Endicott, NY, USA).

## Results

**Figure 8:**
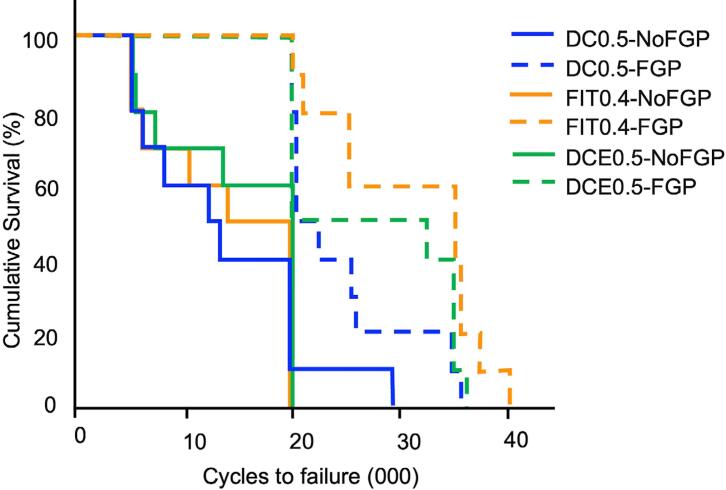
Kaplan-Meier fatigue resistance survival curves (number of cycles under increasing fatigue loads) for all six groups. Solid lines are teeth without fiber glass post (FGP), dashed lines are teeth with FGP

No specimen survived the escalating cyclic loads beyond a mean of 31,000 cycles (spanning 50 N to 200 N). Mean cycle numbers until the failure were used to construct fatigue resistance survival curves (Kaplan-Meier survival estimator) for all 6 groups, shown in Figure 8. Table 1 shows the means and standard errors for the number of cycles to initial and final failure and the pairwise statistical significance level between FGP and No FGP groups.

**Table 1:** Mean cycles endured until initial and final failures with standard errors obtained by Kaplan-Meier survival estimator

Groups	Cycles until initial failure ±SE			Cycles until final failure ±SE		
	No FGP	FGP	p (log-rank test)	No FGP	FGP	p (log-rank test)
DC 0.5	14.570±2.818	26.807±2.045	>0.001*	15.140±2.110	27.460±2.457	>0.001*
FIT 0.4	12.495±2.543	23.926±1.854	>0.001*	14.661±2.350	24.610±1.919	>0.001*
DCE 0.5	13.545±2.756	27.652±1.640	>0.001*	14.496±2.999	30.160±2.275	>0.001*

*DC0.5, root canal prepared using a specific Whitepost System DC 0.5 drill; FIT0.4, root canal prepared using a specific Whitepost System FIT 0.4 drill; DCE 0.5, root canal prepared using a specific Whitepost System DCE 0.5 drill; FGP, presence of fiber glass post cemented in the root canal; No FGP, absence of fiber glass post, only bulk-fill resin-based composite foundation. SE, standard error. p-values of log-rank post hoc pairwise comparisons between initial and final failure within each group. *Statistically significant difference between groups (p<0.05)

The log-rank test showed a significantly higher survival rate of groups with FGP compared with groups without FGP (p<0.001), irrespective of diameter of the FGP and root canal preparation (Table 1 and 2). No significant differences were found among groups with different FGP diameters (p>0.05) or among groups with the bulk-fill RBC foundation and different canal dimension preparations (p>0.05) (Table 2).

**Table 2:** Log-rank (Mantel-COX). p-values of pairwise log-rank post hoc comparisons by Kaplan-Meier survival estimator followed by log-rank test for cycles until failure among all 6 groups (mean values of cycles until failure)

Groups	DC0.5-NoFGP (15.140)	DC0.5-FGP (27.460)	FIT0.4-NoFGP (14.661)	FIT0.4-FGP (24.610)	DCE0.5-NoFGP (14.496)	DCE0.5-FGP (30.160)
DC0.5-NoFGP (15.140)						
DC0.5-FGP (27.460)	>0.001*					
FIT0.4-NoFGP (14.661)	0.719	0.014*				
FIT0.4-FGP (24.610)	>0.001*	0.511	0.039*			
DCE0.5-NoFGP (14.496)	0.732	>0.001*	0.613	0.006*		
DCE0.5-FGP (30.160)	>0.001*	0.282	0.011*	0.064	>0.001*	

*DC0.5, root canal prepared using a specific Whitepost System DC 0.5 drill; FIT0.4, root canal prepared using a specific Whitepost System FIT 0.4 drill; DCE0.5, root canal prepared using a specific Whitepost System DCE 0.5 drill; FGP, presence of fiber glass post cemented in the root canal; No FGP, absence of fiber glass post, only bulk-fill resin-based composite foundation. *Statistically significant difference between groups (p<0.05)

**Figure 9:**
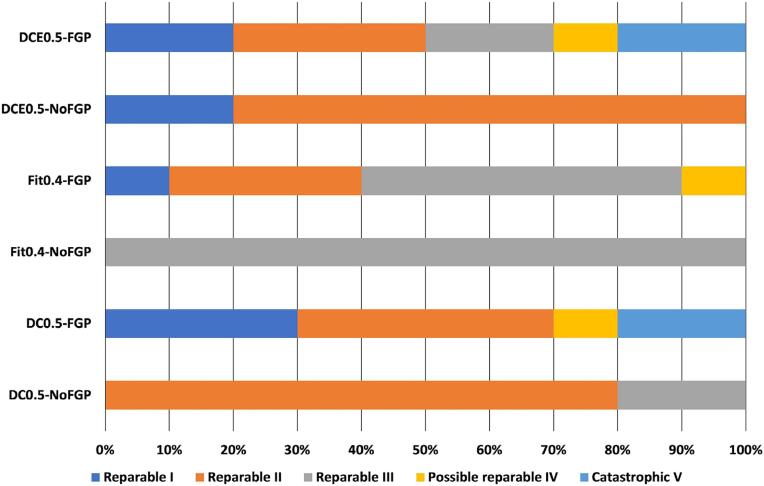
Frequency (percentage) of failure modes for all groups (n=10)

**Figure 10:**
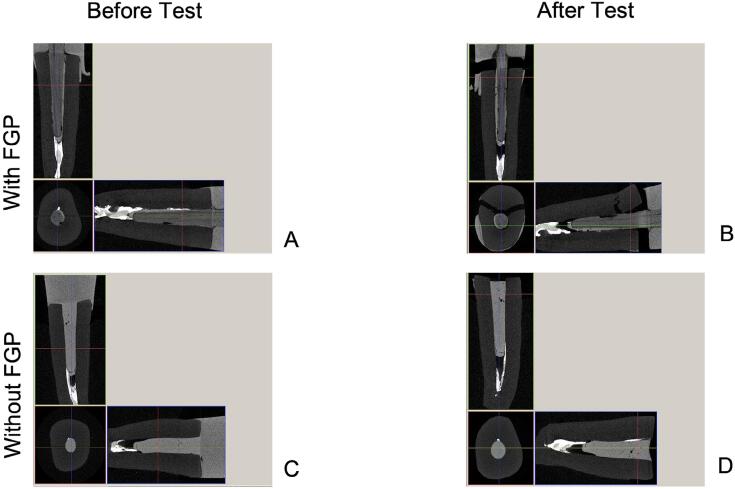
Screenshots of analysis software program (NRecon software program version 1.6.3.3). A and C, Teeth images before the fatigue test. B and D, Teeth images after the fatigue test. A, Resin cement layer with small bubble at apical third. B, fiber glass post (FGP) displacement with root fracture at cervical limit. C, Large bubble at apical third of No-FGP restored tooth. D, Fracture of resin composite retention close to the root canal entrance

The association of transillumination, micro-CT analysis, and digital radiography, demonstrated that almost 70-100% of failures for all groups were repairable. The fracture mode of the groups with a bulk-fill RBC foundation, without FGPs, were always repairable. However, for the groups with FGP DC0.5 and DCE0.5, 20% of the specimens failed catastrophically (Figure 9). The digital radiography (Figure 7) detected the bubbles presence into the RBC and the FGP debonding from the root canal similarly to micro-CT analysis. However, the micro-CT was essential for detecting the severity of the root and FGP fractures (Figure 10). The apical third of the resin cement layer for teeth restored with FGP showed few and small size bubbles.

### Discussion

Severely compromised teeth restored with FGP attained significantly higher cycles to failure compared with teeth with similar restoration but without FGP. Thus, the first null hypothesis was rejected. The FGPs are a more rigid structure than the bulk-fill RBC alone,^25^ and will distribute the incisal edge loading deeper into the root canal. This, alongside modulus properties generally similar to dentin, bonding provided by the treated FGP surface, deep cure of the dual-cure resin cement, and use of a universal adhesive system, has been credited for more favorable stress and strain distributions in the cervical region compared with restorations with only bulk-fill RBC.^6,8,9,31^

The FGP adhesion is thus important for restoring the mechanical behavior of an endodontically treated tooth. When an FGP starts to detach inside the root canal, the risk of root fracture increases significantly.^20^ The micro-CT scans demonstrate that most of the FGP failures started after the posts detached from the root canal dentin. Although the number of FGP fractures was small, creating good bonding inside the root canal remains an important clinical procedure.^35^

The reported survival rates and fracture modes of endodontically treated teeth restored with or without an FGP have not been consistent in the literature. A clinical study, with a mean observation time of 8.8±2.3 years, found that endodontically treated teeth restored with FGP retained restorations had significantly higher survival and success rates (94.3%) than teeth restored without FGP (76.3%).^13^ This agrees with the findings of our *in vitro* study. However, the clinical study also found that the main reason for the failures was root fracture, which were less common in our study. Other studies reported that FGP use did not improve the performance of the restoration of endodontically treated teeth *in vitro*.^3,10,36^ The differences in the results may be due to different experimental designs, such as loading, the absence of simulation of periodontal ligament, the load type used during fracture test, the type of the FGP used, and the amount of remaining coronal structure.^3,10,36^

Besides the presence of an FGP, this study also assessed the effect of the FGP diameter, which had no significant effect on the number of cycles to failure or the fracture mode. Therefore, the second null hypothesis was accepted. Teeth restored with bulk-fill RBC foundation without an FGP also had similar survival results, irrespective of the diameter of the canal preparations. All specimens without FGPs fractured at the RBC foundation, close to the remaining crown. This area has been shown to be a location of higher stress concentrations.^4,5^ Application of an FGP is likely to redistribute these stresses, as changes in the fracture mode distributions in the presence of FGPs suggest.

The number of catastrophic fractures in this study was lower than reported in other studies.^3,33^ Still, the most serious fractures, ‘possibly repairable’ (mode IV) and ‘catastrophic’ (mode V), represented a substantial share at 10 to 30% of the fractures in the FGP restored teeth. Moreover, ‘catastrophic’ fractures occurred both with the largest and the smallest diameter FGPs, consistent with the finding that FGP diameter did not affect the failure mode. In conclusion, using an FGP improved the prognosis in terms of cycles to failure due to the altered stress distribution, but when failure occurred, there was a higher risk of serious damage to the restored teeth.

This study has limitations in application and design. A no-ferrule condition and direct RBC crown were restored with relatively thin FGPs, which can be considered a worst-case situation. However, it shows that even a thin FGP can improve the survival rate of an endodontically treated incisor without ferrule. This study also used bovine teeth instead of human teeth. Bovine teeth were chosen since they improve standardization of specimen size, shape, and properties, which enhances the chance to detect differences among groups that may be difficult to show when using more varied human teeth.^3,9,15^ Another limitation was the simplified loading and fixation conditions. *In vitro* simulations must accelerate the mechanical loading for practical purposes, but most environment driven processes cannot be accelerated. Moreover, variations in load magnitude, angulation, and location, as well as support by a periodontal ligament and surrounding bone, could only be approximated. In this study, fatigue loads were kept constant for fixed periods, and increased stepwise. This accelerated the failure process but cannot be assumed to have a direct correlation with real lifetimes. The support of the root in this study was also approximated, using a polystyrene resin socket, which has elastic properties similar to bone, and by a polyether impression material, which offered some of the flexibility that a periodontal ligament provides for aligning roots under angled incisal edge loading.

## Conclusions

Within the limitations of this *in vitro* study, the following conclusions were reached:

1. Using a FGP increased the survival rate of endodontically treated anterior teeth without ferrule compared with teeth restored with bulk-fill RBC foundation without FGP.
2. Endodontically treated teeth without ferrule restored using FGPs showed similar survival rates and fracture modes irrespective of diameter.
3. Enlarging the root canal preparation, generating a greater volume of RBC foundation, did not affect the survival rate of endodontically treated incisor teeth without ferrule and restored without FGPs.
4. Teeth restored with FGP resulted in fracture modes where the crowns remained mainly in position.
